# Green synthesis of silver and zinc oxide nanoparticles with *Thespesia populnea* extract and investigation of their antioxidant potential against mouse mastitis model

**DOI:** 10.3389/fvets.2025.1521143

**Published:** 2025-03-03

**Authors:** A. Jayasri, P. Eswara Prasad, B. D. P. Kala Kumar, K. Padmaja, P. Shivakumar, B. Anil Kumar, B. Vidya

**Affiliations:** ^1^Department of Veterinary Biochemistry, College of Veterinary Science, Hyderabad, PVNRTVU Telangana, India; ^2^Department of Veterinary Biochemistry, College of Veterinary Science, Tirupati, SVVU, Andhra Pradesh, India; ^3^Department of Veterinary Pharmacology and Toxicology, College of Veterinary Science, Hyderabad, PVNRTVU, Telangana, India; ^4^Department of Veterinary Pharmacology and Toxicology, College of Veterinary Science, Mamnoor, Warangal, PVNRTVU, Telangana, India; ^5^Department of Veterinary Pharmacology and Toxicology, College of Veterinary Science, Korutla, PVNRTVU, Telangana, India; ^6^Department of Livestock Farm Complex, College of Veterinary Science, Hyderabad, PVNRTVU, Telangana, India

**Keywords:** *Thespesia populnea*, TPNS, TPNZ, antioxidant parameters, green synthesis, mice mastitis model

## Abstract

**Introduction:**

Bovine mastitis in dairy cattle is often complicated by antibiotic-resistant bacteria such as *Staphylococcus aureus*. Metal-based nanoparticles, especially plant-mediated nanoparticles have emerged as promising therapeutic tools for treating *S. aureus-*associated mastitis through the intramammary route. In this study, we synthesized, characterized, and assessed the antioxidant activity of *Thespesia populnea* nano silver particles (TPNS) and *Thespesia populnea* nano zinc oxide particles (TPNZ) derived from *Thespesia populnea* leaf extract (TPE). Silver nitrate and zinc acetate were reduced using TPE to synthesize TPNS and TPNZ, which were characterized by Scanning Electron Microscopy (SEM), UV–Visible Spectroscopy, Dynamic Light Scattering (DLS), and Zeta Potential analysis. The antioxidant activity of green-synthesized nanoparticles was evaluated in mastitis-induced mice.

**Methods:**

Forty-eight female Swiss albino mice, 10–15 days of lactation, were divided into six groups (number of mice in each group-8). Group I served as the control, while mastitis was induced in groups II, III, IV, V and VI. Group III received *T. populnea* methanolic leaf extract (TPE); groups IV and V were treated with TPNS and TPNZ respectively; and group VI received Ceftriaxone.

**Results:**

UV–Visible Spectroscopy confirmed the successful reduction of the metal ions to nanoparticles. SEM and DLS analysis revealed agglomerated morphologies with minimal variations in particle size. TPNS had a higher zeta potential than TPNZ, indicating a greater stability in the suspension. Mastitis-induced group showed significantly increased thiobarbituric acid reacting substances (TBARS) levels (*p* < 0.01) and significantly decreased Superoxide dismutase (SOD), Glutathione- S- transferase (GST), catalase (CAT), reduced glutathione (GSH), and glutathione peroxidase (GPx) activities (*p* < 0.01) compared to group I. Improvements were observed in groups IV, VI, V, and III.

**Conclusion:**

The TPNS-treated group (IV) showed the highest restoration of antioxidant activity, followed by the ceftriaxone (VI), TPNZ (V), and TPE-treated groups (III). These findings suggest that phytogenic nanoparticles exhibit higher antioxidant activity than TPE extract alone.

## 1 Introduction

Bovine mastitis is a destructive disease of cattle that causes significant economic losses in the dairy industry (1). *Staphylococcus aureus* is a common cause of bovine mastitis. The disease is linked to oxidative stress from bacterial invasion, as indicated by changes in the oxidative stress parameters in the blood (2, 3). During inflammation, phagocytes produce reactive oxygen species (ROS) that destroy the bacteria (4). Excessive ROS production can overwhelm the antioxidant system and adversely affect the immune system of cows (5). ROS can oxidize macromolecules, such as proteins, lipids, and deoxyribose nucleic acid (DNA), causing oxidative cell damage and altering metabolic pathways (6). Oxidative stress can enhance the adherence of active neutrophils to mammary endothelial cells, worsening inflammation (7). Clinical and subclinical mastitis leads to the release of free radicals and a reduction in the total antioxidant capacity (8). Severe mastitis results in antioxidant imbalance due to excessive peroxynitrite production (9). Evaluating peroxidative damage products (TBARS) and antioxidants, such as glutathione and enzymes (SOD, GPx, and catalase), may serve as markers of oxidative stress and antioxidant status (10). Mastitis alters redox potential, increases oxidative free radicals, and decreases protective antioxidant enzymes (10). In addition to oxidative stress, bacterial infections in mastitis are difficult to combat because of the ability of bacteria to evade the host immune response through biofilms, exotoxins, proteases and bacterial superantigens, and by adhering to mammary epithelial cells (11). *Staphylococcus aureus* induced mastitis poses a significant challenge in the dairy industry because of the ability of bacteria to survive in phagocytes and epithelial cells, rendering antibiotic treatment ineffective (12). Therefore, alternative treatment options are needed. Studies indicate that adequate antioxidant intake in dairy cows enhances immunological functions such as phagocytosis, bacterial killing, and neutrophil oxidative metabolism (13). Recent studies have highlighted that inorganic nanoparticles effectively scavenge reactive oxygen species (14, 15).

Nanomedicine is an emerging field that involves the fabrication of nanoparticles for therapeutic applications (16, 17). Nanoparticles exhibit unique physicochemical properties (17). Various materials, including metals, metal oxides, and silicates, have been used to create nanoparticles (18). Noble metals like copper (Cu), silver (Ag), gold (Au), and titanium (Ti) are commonly used for nanoparticle fabrication (19).

While AgNPs can induce oxidative stress in disease-causing organisms, which indirectly reduces free radical generation, their free radical scavenging activity is attributed to the functional groups present on their surfaces (20). Zinc oxide nanoparticles have demonstrated antioxidant properties in both intracellular and extracellular environments (21). By activating antioxidant enzymes, ZnO nanoparticles reduce the quantity of free radicals intracellularly, whereas their use of electron transfer reduces free radicals in the extracellular environment to perform their free radical scavenging action (22). However, green-synthesized nanoparticles have been found to have higher antioxidant properties, which is attributed to the capping and stabilizing properties of various phytochemicals involved in their production (23).

The production of large quantities of nanoparticles often involves physical techniques that can yield highly pure nanoparticles; however, these techniques typically require expensive equipment, high pressures and temperatures (24, 25), as well as a significant amount of energy. Alternatively, chemical processes such as chemical reduction and electrochemical and sol–gel processes can also be used to create nanoparticles, but these methods may produce hazardous or polluting waste due to the inclusion of toxic reagents or solvents (26, 27).

The synthesis of nanoparticles using green methods primarily involves the incorporation of cell extracts, such as those derived from plants, microorganisms, algae, and fungi, into a substrate without the use of harmful chemicals. The aerial parts of plants, such as the leaves and flowers, are frequently utilized in green synthesis. Numerous researchers have found that proteins and secondary metabolites present in plant extracts serve as reducing and capping agents that promote the production of nanoparticles (28, 29). Phytochemicals, such as vitamins, amino acids, polysaccharides, terpenoids, alkaloids, and other compounds extracted from plants, help in the effective bio-reduction of metal ions during the synthesis of nanoparticles, which exhibit stability and variability in their structure and dimension. Plant components, ranging from leaves to roots, are widely used to produce metal oxide nanoparticles.

*Thespesia populnea* of the Malvaceae family, commonly known as the Indian tulip tree, is widely distributed in the southeastern and coastal forests of India. The bark, blossoms, and leaves of this tree, also known as the portia tree, possess medicinal benefits that can be used to treat skin infections (30). Research has shown that *T. populnea* leaves contain flavonoids, tannins, saponins, terpenoids, polyphenols, glycosides, alkaloids, quercetin, phytosterols, lupeol, and rutin (31, 32). The phytochemicals found in *T. populnea* have been shown to possess anti-inflammatory, anti-diarrheal, antibacterial, antifungal, and haemostatic properties so it is used in traditional medicinal systems like Sidha and Ayurveda especially the bark and leaves are often used in decoctions or poultices, while the fruits and seeds are be used in oil preparations (33, 34). However, studies examining the effectiveness of *T. populnea* herbal extract in eliminating oxidative stress related to bacterial mastitis using metal nanoparticles are limited. Considering this, the current study aimed to investigate the green synthesis and characterization of Ag and ZnO nanoparticles from *T. populnea* leaf extract, as well as the antioxidant activity of these nanoparticles in the treatment of mastitis in a murine model.

This study focuses on the synthesis and characterization of silver (AgNPs), ZnO nanoparticles derived from *Thespesia populnea* extract using green synthesis approach. The main objective is to evaluate the antimicrobial activity of the nanoparticles against *Staphylococcus aureus induced mouse mastitis model*, assess their antioxidant properties *in vivo*, and investigate their potential for reducing oxidative stress in mastitis model. The study did not include long-term toxicity assessments and the plant extract was sourced during a single season, which may limit the seasonal variability of its bioactive compounds.

## 2 Materials and methods

The experiment was conducted at the Department of Veterinary Biochemistry, College of Veterinary Science, Rajendranagar, Telangana, India, using *T. populnea* leaves collected from Andhra Pradesh, India which were harvested during the flowering season (February to March). Higher concentrations of bioactive compounds were found during this period in *Thespesia populnea.*

### 2.1 Preparation of *T. populnea* methanolic leaf extract

Hundred gram of dried, coarsely powdered *T. populnea* leaves was soaked in 95% methanol for 72 h with intermittent mixing. The concentrated filtrate was air-dried and the percentage yield was calculated after weighing.

### 2.2 Synthesis of TPNS

Ninety milliliter of 0.1 M silver nitrate solution was added to 10 mL of 2% *T. populnea* methanolic leaf extract at 95°C with vigorous stirring. The color change of the solution from pale yellow to brown indicates the formation of TPE-mediated AgNPs.

### 2.3 Synthesis of TPNZ

Four milliliter of TPE was added dropwise to 0.5% zinc acetate, and the solution was mixed using a magnetic stirrer for 10 min. The pH was adjusted to 12 using 2 M NaOH, resulting in a white crystalline ZnO precipitate, which was repeatedly washed, filtered, and dried at 60°C to obtain ZnO nanoparticles.

### 2.4 Scanning electron microscopy

Morphology of TPNS and TPNZ nanoparticles was determined by SEM machine (JEOL JSM—5,600, Japan) operating in high vacuum mode with an acceleration voltage of 15 kV.

### 2.5 Dynamic light scattering analysis

The particle velocity distribution was assessed by measuring the dynamic fluctuations in the light-scattering intensity, and the Stokes-Einstein equation was used to determine the hydrodynamic radius or diameter, with measurements conducted using a Nanopartica SZ-100 instrument (Horiba, Japan).

### 2.6 Zeta potential

Zeta potential provides the net surface charge of the nanoparticles, as determined by Kim et al. (35).

### 2.7 Animals

Female albino mice (25–35 g) were sourced from M/s. Jeeva Life Sciences, Hyderabad, Telangana, India and were approved by the Institutional Animal Ethics Committee (I/2018-3/IAEC/C.V.Sc., Hyd).

### 2.8 Experimental design

Forty-eight lactating female *Swiss Albino* mice (10–15 days postpartum) weighing 35–40 g were randomly divided into six groups (*n* = 8). Group I was the control group. After anaesthesia using a mixture of ketamine and xylazine at the rate of 87 and 13 mg/kg of body weight, respectively, mastitis was induced in groups II to VI via intramammary inoculation of 20 μL of *S. aureus* (4.0 × 10^4^ C.F.U.) isolated from a field strain isolated from bovine mastitis in Left 4th teat (36) with a 33-gauge hamilton blunt needle after exposing the teat canal by cutting the end of the teat under a binocular microscope. The antibiotic susceptibility profile of the *S. aureus* strain was determined prior to the study using the disc diffusion method. The strain was tested for sensitivity to ceftriaxone using a ceftriaxone disc (30 μg), and the zone of inhibition was measured. The highest zone of inhibition was found to be against ceftriaxone followed by tetracycline, gentamycin, O floxacin and streptomycin.

Later, the mice were administered butorphenol at a rate of 3–5 mg/kg body weight to prevent post-inoculation trauma. The CPCSEA guidelines were followed during the procedure. Six hours post-inoculation, Group I received PBS; Groups III, IV, V, and VI were intramammary administered 20 μL each of TPE (in 1% aqueous DMSO), TPNS, TPNZ, and Ceftriaxone (Intacef-4, INTAS Pharmaceuticals Limited, INDIA) into L4. The induction of mastitis in the mice was confirmed by observing characteristic signs of inflammation (swelling, redness, and discharge) at the site of infection within first 24 h. After 48 h of inocculation, the signs were more pronounced and the mice were anesthetized with ketamine and euthanized using Co_2_ chamber. Blood collected via cardiac puncture was stored in heparin-coated tubes for oxidative stress and antioxidant analysis. To evaluate oxidative stress and antioxidant parameters, whole blood was used to estimate GSH (37), and hemolysate was prepared to assess TBARS (38), SOD (39), CAT (40), GPx (41), and GST (42).

The green synthesized nanoparticles were characterized using SEM, DLS, and UV–Vis spectroscopy. *In vitro* antimicrobial testing was performed against *Staphylococcus aureus*, while *in vivo* antioxidant effects were assessed in murine mastitis model. No seasonal variation of the plant extract was considered, and the study did not include investigations into chronic toxicity.

### 2.9 Statistical analysis

The data obtained from the experimental animals of different treatment groups were tabulated and analyzed to determine the significance among the experimental groups according to the procedures of Snedecor and Cochran (43) using a statistical package for social sciences (SPSS – 20 software, IBM, United States). Statistical significance was analyzed using one-way factorial analysis of variance (ANOVA) and evaluated using Duncan’s multiple comparison test. The significance level was set at *p* < 0.01. Data are expressed as mean ± standard error (SE).

## 3 Results

**Figure 1:**
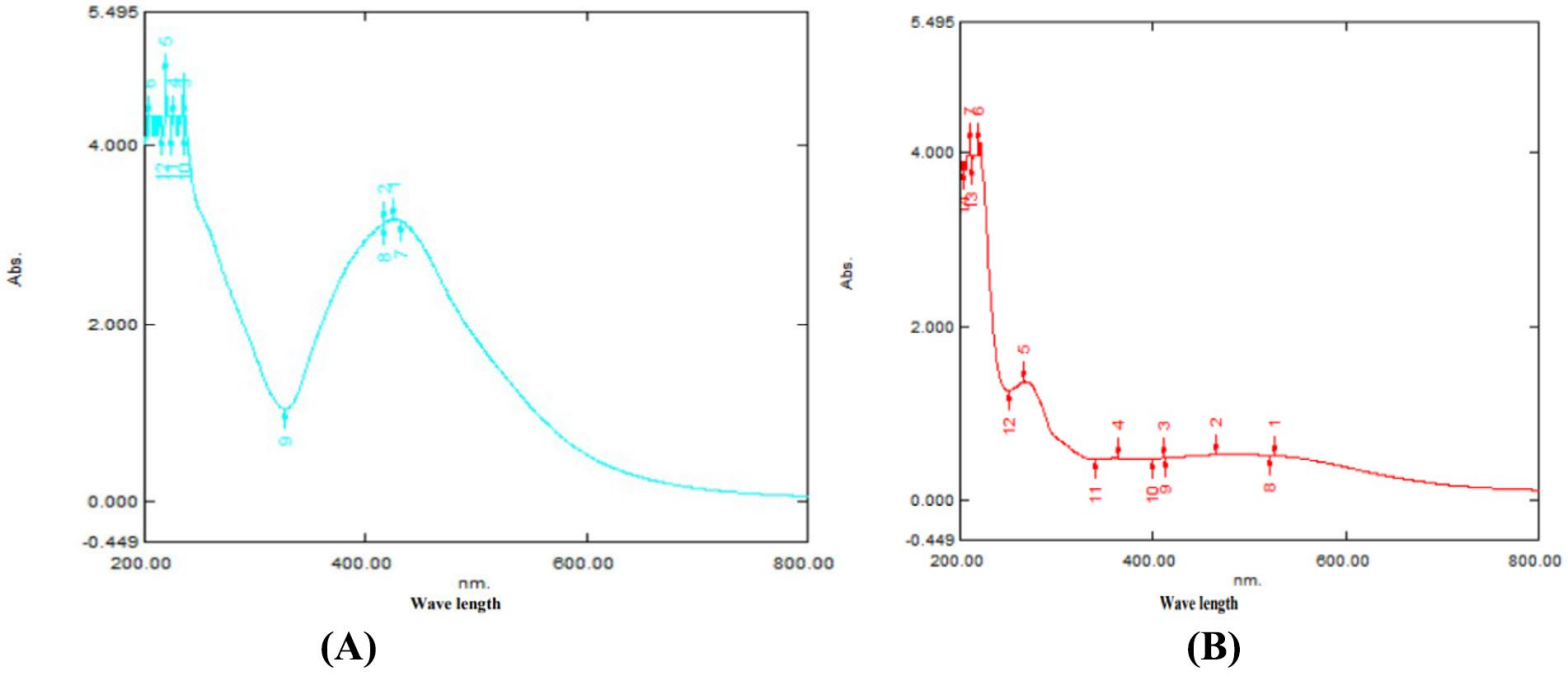
**(A)** UV-Visible spectrum of TPNS. **(B)** TPNZ nanoparticles.

Synthesis and Characterization of Nanoparticles UV–VIS Analysis: Figures 1A,B display the UV–visible absorption spectra of the TPNS and TPNZ particles, respectively. TPNS particles exhibited a maximum absorbance peak at 421 nm, confirming the bioreduction of Ag^+^ to Ag (0). The absorption spectrum of the TPNZ particles, recorded between 200 and 800 nm, showed a peak at 260 nm, indicating the formation and stability of ZnO nanoparticles.

### 3.1 Characterization of *T. populnea* methanolic extract mediated nanoparticles using UV–visible spectroscopy

The reduction of pure nano ions was monitored by measuring the UV–visible spectrum of the reaction medium after 5 h, with the sample diluted in distilled water, using a UV–Visible Spectrophotometer (Spectrophotometer UV–VIS spectrophotometer UV-2450, Shimadzu, Japan).

### 3.2 Scanning electron microscopy analysis

**Figure 2:**
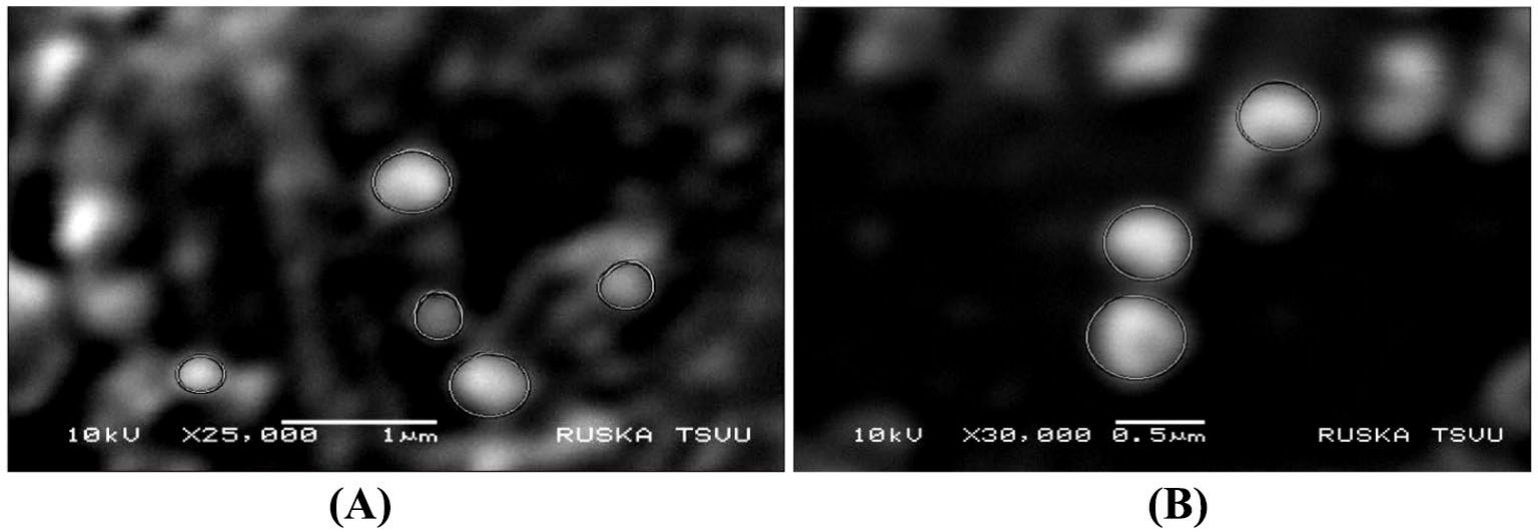
**(A)** SEM image analysis of TPNS particles. **(B)** TPNZ particles.

SEM analysis of the TPNS particles, depicted in Figure 2A, shows electron-dense and elliptical-to-spherical nanoparticles arranged in clusters. The SEM analysis of the TPNZ particles (Figure 2B) showed that the spherical particles were uniformly distributed.

### 3.3 DLS technique

**Figure 3:**
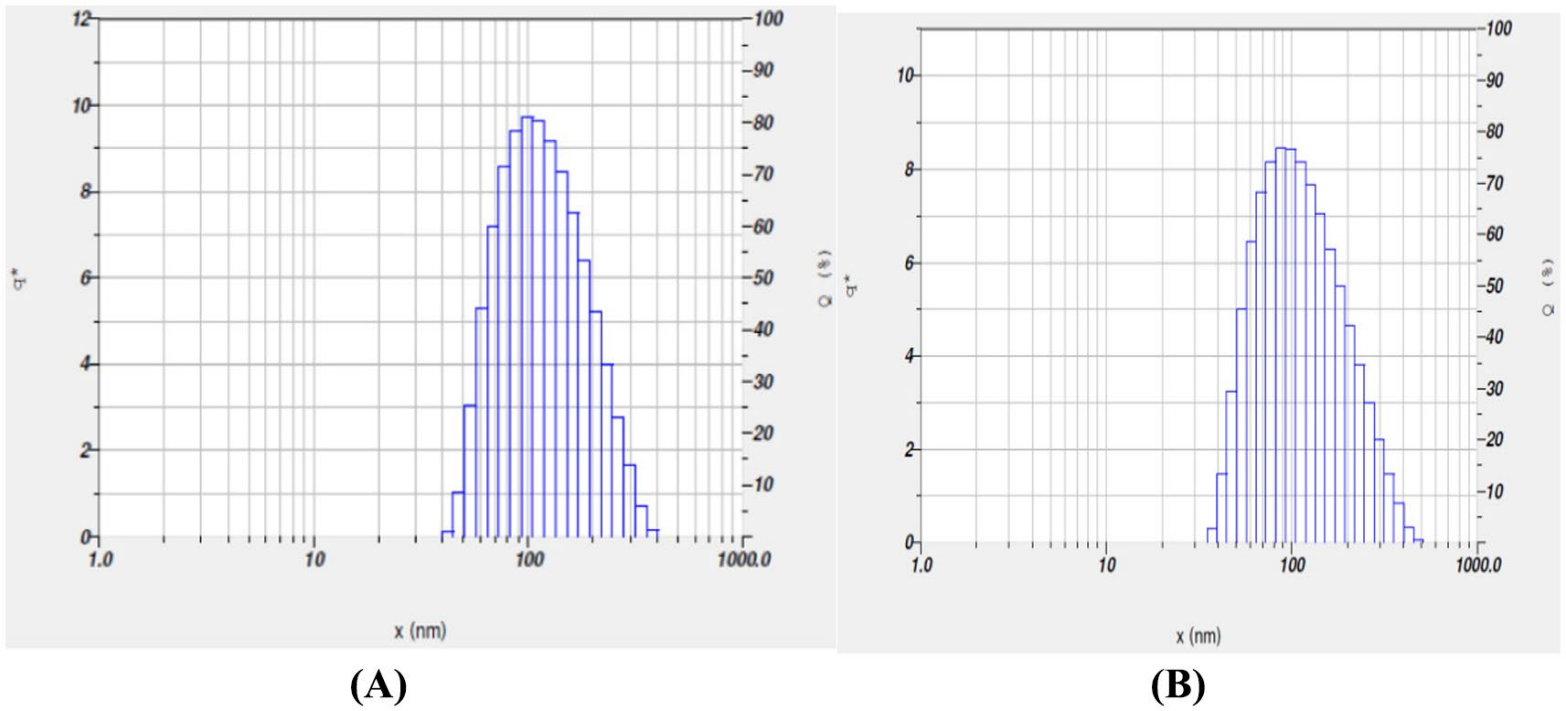
**(A)** Dynamic Light Scattering (DLS) analysis of TPNS nanoparticles. **(B)** Dynamic Light Scattering (DLS) analysis of TPNZ nanoparticles.

DLS technique was used to determine the hydrodynamic diameter of the nanoparticles. The measurements revealed that the TPNS particles (Figure 3A) had a size of 99 nm, while the TPNZ particles (Figure 3B) exhibited a size of 87.7 nm.

### 3.4 Zeta potential

**Figure 4:**
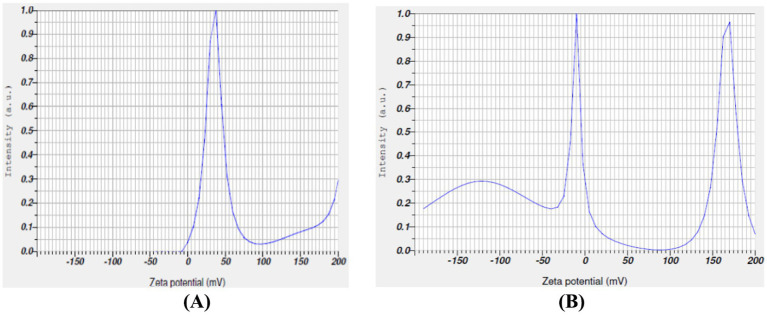
**(A)** Zeta potential measurement of TPNS nanoparticles. **(B)** Zeta potential measurement of TPNZ nanoparticles.

The zeta potential for *T. populnea*-mediated nano-silver nanoparticles was measured as 90.5 mV (Figure 4A) with an electrophoretic mobility (mean) of −0.000700 cm^2^ /Vs. The zeta potential and electrophoretic mobility (Figure 4B) of *T. populena* mediated nano ZnO particles were found to be 48.5 mv and 000376 cm^2^ /*Vs*, respectively.

### 3.5 Evaluation of oxidative stress and antioxidant parameters

Oxidative stress marker assays confirmed the antioxidant efficacy of the synthesized TPNS and TPNZ particles. Group II exhibited significantly elevated TBARS levels and reduced SOD, CAT, GSH, GPx, and GST activities (*p* < 0.01) compared with the other groups. No significant differences were observed in TBARS and GSH activities between groups IV and I. Similarly, SOD and GST activities did not differ significantly between groups III and V. Table 1 shows no significant difference in GPx activity between Groups V and VI. TBARS levels in Groups VI, V, and III were significantly lower (*p* < 0.01) than those in Group II. The activities of SOD, CAT, GSH, GPx, and GST were significantly increased (*p* < 0.01) in groups IV, VI, V, and III, respectively, compared with those in group II.

**Table 1:** Mean (±SE) values of oxidative stress and Anti-oxidant parameters in blood of different experimental groups.

Oxidative stress and anti-oxidant parameters	Group I	Group II	Group III	Group IV	Group V	Group VI
TBARS (nano moles/gm of Protein)	0.198^e^ ± 0.004	0.365^a^ ± 0.005	0.307^b^ ± 0.007	0.202^e^ ± 0.005	0.281^c^ ± 0.008	0.251^d^ ± 0.004
SOD (units/mg of protein)	25.16^a^ ± 0.87	12.84^e^ ± 0.36	18.12^d^ ± 0.31	23.08^b^ ± 0.45	19.13^d^ ± 0.38	21.35^c^ ± 0.29
CAT (μ moles of H2O2 utilized /min/mg of protein)	133.28^a^ ± 0.43	107.91^f^ ± 0.39	118.19^e^ ± 0.43	131.73^b^ ± 0.45	122.80^d^ ± 0.38	126.25^c^ ± 0.48
GSH (μmoles/mg of protein)	5.58^a^± 0.02	2.33^e^± 0.03	3.53^d^± 0.02	5.54^a^ ± 0.03	4.46^c^ ± 0.02	4.76^b^ ± 0.01
GPx (units/gm of protein)	29.93^a^± 0.32	15.56^e^± 0.49	20.28^d^± 0.48	27.23^b^ ± 0.51	22.24^c^ ± 0.39	23.4^c^ ± 0.49
GST (μmoles of CDNB-GSH conjugate formed/min/mg of protein)	3.11^a^± 0.18	0.88^e^± 0.02	1.94^d^± 0.08	2.89^b^ ± 0.06	2.05^d^ ± 0.1	2.43^c^ ± 0.05

The different superscripts in the column are the mean values which differ significantly (*P* < 0.01).

## 4 Discussion

Green synthesis of nanoparticles, leveraging various phytochemicals in plant extracts, is biocompatible and environmentally friendly, making it efficient for large-scale biomedical applications (44). In this study silver and ZnO nanoparticles were synthesized using *T. populnea* methanolic leaf extract and characterized by UV–VIS analysis, SEM, DLS, and Zeta potential measurements. The addition of 1 mM silver nitrate and zinc acetate to *T. populnea* leaf extract resulted in a color change, confirming the production of TPNS and TPNZ (45, 46). UV–VIS spectroscopy indicated peaks at 421 nm and 260 nm for TPNS and TPNZ, respectively, suggesting bioreduction of aqueous silver ions (Ag+) upon exposure to plant extracts. Phytochemicals in *T. populnea* leaf extracts facilitate the transformation of silver ions into metallic nanoforms (47). Previous studies have shown peaks around 420 nm for *T. populnea*-synthesized silver nanoparticles (48) and 295 nm for TPNZ (49), while ZnO nanoparticles from *Deverra tortuosa* and the aqueous extract exhibited peaks in the 200–800 nm range (50), which is consistent with the findings of this studyTPNS and TPNZ particles were further characterized using SEM to examine their morphologies and structures. The SEM image analysis in this study revealed the formation of elliptical to spherical agglomerated TPNS, consistent with the findings of Bhuyar et al. (51) and Widatalla et al. (52) using *Padina* sp. and green tea leaf extracts. SEM images of TPNZ showed uniformly distributed spherical particles, aligning with results from Yedurkar et al. (53) and Muhammad et al. (54), who synthesized spherical ZnO nanoparticles using *Ixora coccinea* and *Papaver somniferum* leaf extracts, respectively. DLS, a technique for measuring particle size through laser beam analysis of Brownian motion in suspension, revealed sizes of 99 nm for TPNS and 87.7 nm for TPNZ. Similar diameters were reported for TPNS synthesized from *Rizophora apiculata* (99 nm) (55). Comparable sizes of 70 and 100 nm have reported for TPE-mediated silver nanoparticles (48) and *M. oleifera* seed extract-mediated silver nanoparticles (56). Sundrarajan et al. (57) reported a size of 100 nm for *Pongamia pinnata* leaf extract-mediated nano ZnO particles via DLS, while Shukla et al. (58) showed a size range of 76.2 to 183.8 nm for Zinc oxide nanoparticles synthesized from *Aspergillus niger*.

The zeta potential method is crucial for estimating the surface charge of nanoparticles, which is essential for their characterization and understanding the physical stability of nanosuspensions (19). Studies (59) have indicated that stable particles have a zeta potential of ≥ + 30 mV or ≤ −30 mV. A positive charge value of +37.4 mv of zeta potential for silver nanoparticles synthesized using *Morus alba* leaf extract were reported by Das et al. (60).

TPNS displays higher zeta potential than TPNZ, suggesting that TPE can effectively mediate nano-silver compared to nano ZnO particles.

**Figure 5:**
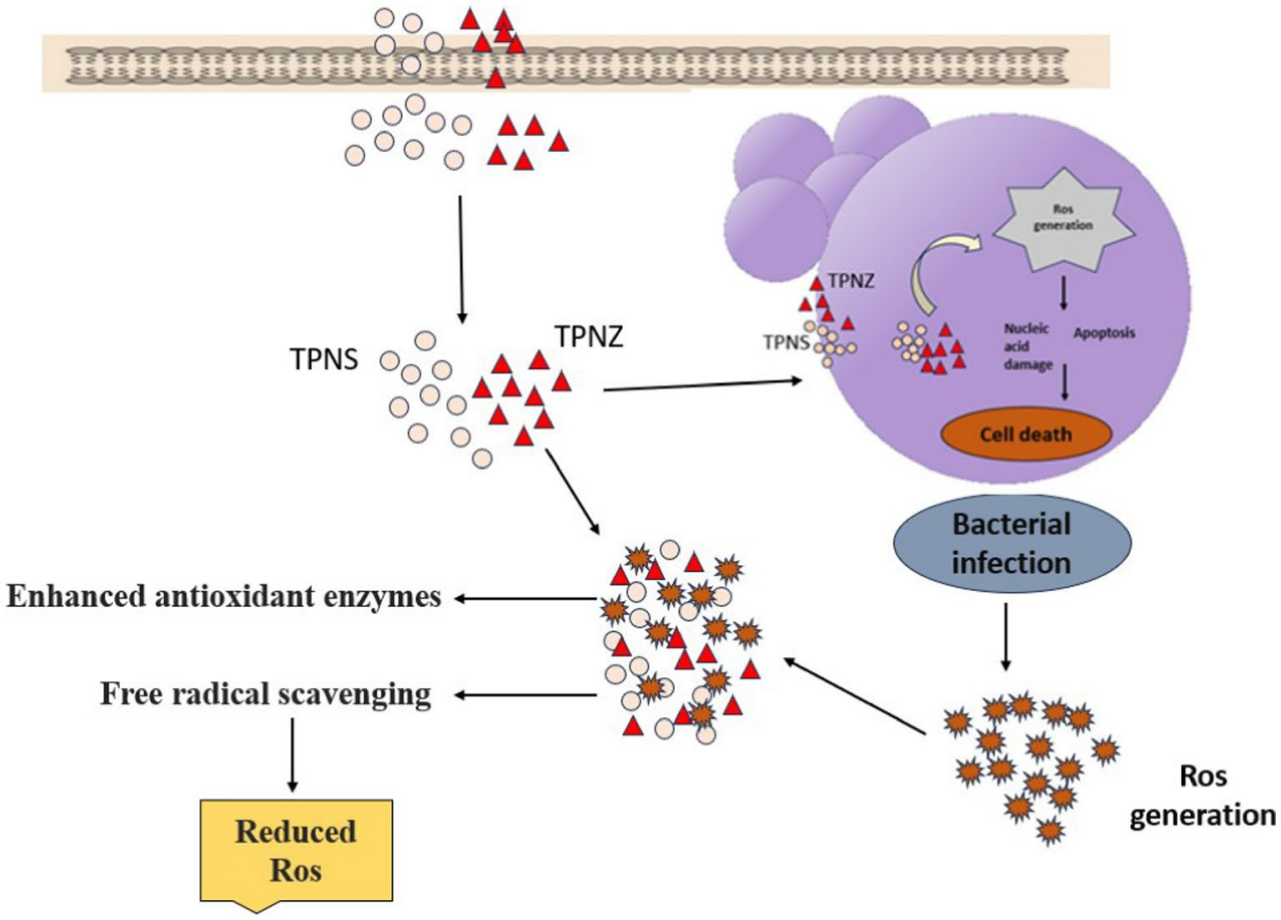
Schematic diagram representing the antioxidant activity of silver and zinc oxide nanoparticles.

Reactive oxygen species (ROS) are the natural byproducts of cellular metabolism. Oxidative stress occurs when ROS production exceeds the antioxidant defense capacity (61). In dairy cattle, both clinical and subclinical mastitis increase free radical production, increase total oxidant capacity, and reduce total antioxidant capacity (8). Lipid peroxidation products, particularly polyunsaturated fatty acids susceptible to free radical attack, are commonly used as oxidative stress markers, with TBARS being a widely recognized indicator (62). The elevated TBARS levels in Group II indicated oxidative stress. Among the treated groups, higher restoration of TBARS values was observed in group IV, followed by VI, V, and III, suggesting that TPNS had a stronger antioxidant effect than ceftriaxone, TPNZ, and TPE alone. Siddique and Al-Samman (63) observed a similar decrease in TBARS with *Delphinium denudatum* wall. Root extract-mediated AgNPs in mice with nephrotoxicity. However, aloin-mediated nano-silver and 11-*α*-keto boswellic acid-mediated nano-silver did not effectively exert antioxidant effects (64, 65). Kiyani et al. (66) reported a significant (*p* < 0.01) reduction in TBARS, approaching control values, in gout-affected mice treated orally with nano ZnO (Figure 5).

Endogenous antioxidants include enzymes such as superoxide dismutase (SOD), catalase (CAT), glutathione peroxidase (GPx), and reductase, play a crucial role in mitigating oxidative stress. The activities of these enzymes increase during oxidative stress to neutralize excess free radicals. During mastitis, PMNs are activated and generate reactive oxygen species (ROS) such as H_2_O_2_, superoxide anions, hydroxyl radicals, and halogen reactive species, partially reducing O_2_ and lowering antioxidant enzyme levels (67). In this study, the activities of SOD, CAT, GSH, GPx, and GST significantly decreased (*p* < 0.01) in the blood of mastitis-induced mice, indicating oxidative stress, which is consistent with the findings of Chinchali and Kaliwal (68). The reduced enzyme activities in the mastitis-affected group were normalized more effectively in group IV, followed by groups VI, V, and III, suggesting a higher antioxidant activity of TPNS, likely due to its increased free radical scavenging capacity. GPx activity was nearly restored in mice treated with *Rhizophora apiculata*-derived AgNPs in hepatotoxin-induced liver damage (69). Yadav et al. (70) reported a significant (*p* < 0.001) increase in SOD, catalase, and GPx activity in the granulation tissue of rats treated with *T. portulacastrum*-mediated nano ZnO compared to untreated rats in an induced wound model.

Suresh et al. (71) reported the antioxidant activity of *Cassia fistula*-mediated nano ZnO in *in vitro* assays. Ilavarasan et al. (72) and Pandanaboina et al. (73) demonstrated the antioxidant activity of TPE in rats with carbon tetrachloride-induced liver injury and alcohol-induced hepato-renal injury, respectively. Chaitanya et al. (64) observed improved glutathione levels with aloin-mediated silver nanoparticles in mastitis-induced mice. Jacob and Rajiv (74) showed that *Curcuma longa*-mediated nano ZnO particles possess free radical scavenging abilities through *in vitro* assays. ZnO nanoparticles enhance antioxidant enzyme activities, reduce free radical levels (OH., O_2_., H_2_O_2_), and scavenge free radicals by electron transfer. Silver nanoparticles synthesized via plant extract phytochemicals efficiently reduce reactive oxygen species (ROS) and protecting biomolecules (75). Biosynthesized AgNPs exhibit superior antioxidant activity compared to extracts alone because of their large surface area, which enhances bioactive chemical adsorption (76). Our findings align with recent literature suggesting that AgNPs, particularly when interacting with antioxidants or phytochemicals, may offer therapeutic benefits. Specifically, studies have demonstrated that AgNPs act as catalysts in antioxidant reactions or facilitate cellular repair under controlled conditions, such as low doses or in combination with herbal compounds (77). TPE can be mediated with nano silver and nano ZnO particles, exerting more effective antioxidant effects than the methanolic extract alone.

## 5 Conclusion

This study explores the green synthesis and characterization of nanoparticles, specifically silver and ZnO nanoparticles, using TPE-mediated leaf extract. In the present study, it was shown that the overall antioxidant activity of TPNS was higher than that of ceftriaxone, TPNZ, and TPE indicating that biologically synthesized nanoparticles are more potent than the TPE extract alone, likely due to the combined antioxidant effect of phytochemicals and nanoparticles. Further safety studies are necessary for the upscaling and potential parental use of TPE-mediated nanoparticles as effective antioxidant agents.

### 5.1 Limitations of the study

The study has certain limitations, including the lack of seasonal variation analysis, as the plant extract was sourced during a specific season, which may affect the reproducibility due to changes in bioactive compound composition. Additionally, the analysis of oxidative stress was limited to serum antioxidant parameters, and other organs such as liver and kidneys were not analyzed for oxidative stress. The *in vivo* studies were not done as it was beyond the objective of the experiment. Furthermore, the long-term stability of the nanoparticles was not assessed.

## Data Availability

The original contributions presented in the study are included in the article/supplementary material, further inquiries can be directed to the corresponding author.

## References

[1] Ruegg PL. A 100-year review: mastitis detection, management and prevention. J Dairy Sci. (2017) 100:10381–97. doi: 10.3168/jds.2017-13023, PMID: 29153171

[2] Ranjan R Swarup D Naresh R Patra RC. Enhanced erythrocytic lipid peroxides and reduced plasma ascorbic acid, and alteration in blood trace elements level in dairy cows with mastitis. Vet Res Commun. (2005) 29:27–34. doi: 10.1023/B:VERC.0000046740.59694.5d, PMID: 15727289

[3] Kizil O Akar YAŞAR Saat N Kizil M Yuksel M. The plasma lipid peroxidation intensity (MDA) and chain-breaking antioxidant concentrations in the cows with clinic or subclinic mastitis. Rev Med Vet. (2007) 158:529–33.

[4] Sies H Mailloux RJ Jakob U. Fundamentals of redox regulation in biology. Nat Rev Mol Cell Biol. (2024) 25:701–19. doi: 10.1038/s41580-024-00730-2, PMID: 38689066 PMC11921270

[5] Sordillo LM Aitken SL. Impact of oxidative stress on the health and immune function of dairy cattle. Vet Immunol Immunopathol. (2009) 128:104–9. doi: 10.1016/j.vetimm.2008.10.305, PMID: 19027173

[6] Basirico L Vetturini T Bernabucci U. Etiology of oxidative stress in dairy cow In: Gross JJ, editor. Production diseases in farm animals. Cham: Springer (2024). 99–114.

[7] Wang X Ban C Li JX Luo QY Qin JX Xu YQ. Differentially expressed genes and signalling pathways regulated by high selenium involved in antioxidant and immune functions of goats based on transcriptome sequencing. Int J Mol Sci. (2023) 24:1124. doi: 10.3390/ijms24021124, PMID: 36674636 PMC9864924

[8] Atakisi O Oral H Atakisi E Merhan O Pancarci SM Ozcan A. Subclinical mastitis causes alterations in nitric oxide, total oxidant and antioxidant capacity in cow milk. Res Vet Sci. (2010) 89:10–3. doi: 10.1016/j.rvsc.2010.01.008, PMID: 20132956

[9] Chaiyotwittayakun A Erskine RJ Bartlett PC Herdt TH Sears PM Harmon RJ. The effect of ascorbic acid and L-histidine therapy on acute mammary inflammation in dairy cattle. J Dairy Sci. (2002) 85:60–7. doi: 10.3168/jds.S0022-0302(02)74053-8, PMID: 11860122

[10] Suresh MK Vasudevan AK Biswas L Biswas R. Protective efficacy of alum adjuvanted amidase protein vaccine against *Staphylococcus aureus* infection in multiple mouse models. J Appl Microbiol. (2022) 132:1422–34. doi: 10.1111/jam.15291 34487603

[11] Peralta OA Carrasco C Vieytes C Tamayo MJ Muñoz I Sepulveda S. Safety and efficacy of a mesenchymal stem cell intramammary therapy in dairy cows with experimentally induced *Staphylococcus aureus* clinical mastitis. Sci Rep. (2020) 10:2843. doi: 10.1038/s41598-020-59724-7, PMID: 32071371 PMC7028716

[12] Pereira UP Oliveira DGS Mesquita LR Costa GM. Pereira LJ efficacy of *Staphylococcus aureus* vaccines for bovine mastitis: a systematic review. Vet Microbiol. (2011) 148:117–24. doi: 10.1016/j.vetmic.2010.10.003, PMID: 21115309

[13] Mion B Ogilvie L Van Winters B Spricigo JF Anan S Duplessis M. Effects of replacing inorganic salts of trace minerals with organic trace minerals in the pre-and postpartum diets on mineral status, antioxidant biomarkers, and health of dairy cows. J Ani Sci. (2023) 101:skad041. doi: 10.1093/jas/skad041, PMID: 36734127 PMC9994592

[14] Du L Suo S Wang G Jia H Liu KJ Zhao B. Mechanism and cellular kinetic studies of the enhancement of antioxidant activity by using surface-functionalized gold nanoparticles. Chem Eur. (2013) 19:1281–7. doi: 10.1002/chem.201203506, PMID: 23229373

[15] Ramamurthy CH Padma M Mareeswaran R Suyavaran A Kumar MS Premkumar K. The extra cellular synthesis of gold and silver nanoparticles and their free radical scavenging and antibacterial properties. Colloids Surf B Biointerfaces. (2013) 102:808–15. doi: 10.1016/j.colsurfb.2012.09.025, PMID: 23107960

[16] Dahl JA Maddux BL Hutchison JE. Toward greener nanosynthesis. Chem Rev. (2007) 107:2228–69. doi: 10.1021/cr050943k, PMID: 17564480

[17] Buzea C Pacheco II Robbie K. Nanomaterials and nanoparticles: sources and toxicity. Biointerphases. (2007) 2:MR17–71. doi: 10.1116/1.2815690, PMID: 20419892

[18] Ahmed S Ahmad M Swami BL Ikram S. A review on plants extract mediated synthesis of silver nanoparticles for antimicrobial applications: a green expertise. J Adv Res. (2016) 7:17–28. doi: 10.1016/j.jare.2015.02.007, PMID: 26843966 PMC4703479

[19] Narayanan M Srinivasan B Sambantham MT Al-Keridis LA AL-mekhlafi FA. Green synthesizes and characterization of copper-oxide nanoparticles by *Thespesia populnea* against skin-infection causing microbes. J King Saud Univ Sci. (2022) 34:101885–8. doi: 10.1016/j.jksus.2022.101885, PMID: 39811820

[20] Keshari AK Srivastava R Singh P Yadav VB Nath G. Antioxidant and antibacterial activity of silver nanoparticles synthesized by *Cestrum nocturnum*. J Ayurveda Integr Med. (2020) 11:37–44. doi: 10.1016/j.jaim.2017.11.003, PMID: 30120058 PMC7125370

[21] Jha S Rani R Singh S. Biogenic zinc oxide nanoparticles and their biomedical applications: a review. J Inorg Organomet Polym Mater. (2023) 33:1437–52. doi: 10.1007/s10904-023-02550-x, PMID: 37359387 PMC10118236

[22] Das D Nath BC Phukon P Dolui SK. Synthesis of ZnO nanoparticles and evaluation of antioxidant and cytotoxic activity. Colloids Surf B Biointerfaces. (2013) 111:556–60. doi: 10.1016/j.colsurfb.2013.06.041, PMID: 23891844

[23] Doroteo V Díaz C Terry C Rojas R. Phenolic compounds and antioxidant activity in vitro of 6 Peruvian plants. Rev Soc Quím Perú. (2013) 79:13–20.

[24] Zhang XF Liu ZG Shen W Gurunathan S. Silver nanoparticles: synthesis, characterization, properties, applications, and therapeutic approaches. Int J Mol Sci. (2016) 17:1–34. doi: 10.3390/ijms17091534, PMID: 27649147 PMC5037809

[25] Yaqoob AA Umar K Ibrahim MNM. Silver nanoparticles: various methods of synthesis, size affecting factors and their potential applications–a review. Appl Nanosci. (2020) 10:1369–78. doi: 10.1007/s13204-020-01318-w

[26] Tran QH Le AT. Silver nanoparticles: synthesis, properties, toxicology, applications and perspectives. Adv Nat Sci Nanosci Nanotechnol. (2013) 9:1–20. doi: 10.1088/2043-6254/aad12b, PMID: 39717972

[27] Krutyakov YA Kudrinskiy AA Olenin AY Lisichkin GV. Synthesis and properties of silver nanoparticles: advances and prospects. Russ Chem Rev. (2008) 77:233–57. doi: 10.1070/RC2008v077n03ABEH003751

[28] Rajeshkumar S Bharath LV. Mechanism of plant-mediated synthesis of silver nanoparticles–a review on biomolecules involved, characterisation and antibacterial activity. Chem Biol Interact. (2017) 273:219–27. doi: 10.1016/j.cbi.2017.06.019, PMID: 28647323

[29] Rónavári A Igaz N Adamecz DI Szerencsés B Molnar C Kónya Z. Green silver and gold nanoparticles: biological synthesis approaches and potentials for biomedical applications. Molecules. (2021) 26:1–39. doi: 10.3390/molecules26040844, PMID: 33562781 PMC7915205

[30] Vishwakarma A Arora P Dhobi M. *Thespesia populnea*: an Ethnomedicinal, phytochemical and pharmacological review. Nat Prod J. (2022) 12:2–12. doi: 10.2174/2210315510999201210142313, PMID: 39791164

[31] Nagappa AN Cheriyan B. Wound healing activity of the aqueous extract of *Thespesia populnea* fruit. Fitoterapia. (2001) 72:503–6. doi: 10.1016/s0367-326x(01)00275-1, PMID: 11429243

[32] Muthukumar S Veerappa NS. Phytochemical analysis in the root and leaf of *Thespesia populnea* (Linn) Soland ex correa. J Pharmacogn Phytochem. (2018) 7:414–7.

[33] Vasudevan M Parle M. Pharmacological actions of *Thespesia populnea* relevant to Alzheimer's disease. Phytomedicine. (2006) 13:677–87. doi: 10.1016/j.phymed.2006.01.007, PMID: 16860552

[34] Lindamulage IKS Soysa P. Evaluation of anticancer properties of a decoction containing *Adenanthera pavonina* L. and *Thespesia populnea* L. BMC Complement Altern Med. (2016) 16:70. doi: 10.1186/s12906-016-1053-9, PMID: 26896952 PMC4761162

[35] Kim JS Kuk E Yu KN Kim JH Park SJ Lee HJ. Antimicrobial effects of silver nanoparticles. Nanomedicine. (2007) 3:95–101. doi: 10.1016/j.nano.2006.12.001, PMID: 17379174

[36] Peng J Lu Q Yuan L Zhang H. Synthetic cationic lipopeptide can effectively treat mouse mastitis caused by *Staphylococcus aureus*. Biomedicines. (2023) 11:1188. doi: 10.3390/biomedicines11041188, PMID: 37189805 PMC10136272

[37] Atakisi O Dalginli KY Gulmez C Kalacay D Atakisi E Zhumabaeva TT. The role of reduced glutathione on the activity of adenosine deaminase, antioxidative system, and aluminum and zinc levels in experimental aluminum toxicity. Biol Trace Elem Res. (2023) 201:4429–36. doi: 10.1007/s12011-022-03503-0, PMID: 36456741

[38] Dar MA Siddiqui NA Raja WY Mir PA Qadir I Masoodi MH. Amelioration of experimental hyperlipidemia in rats by *Portulaca oleracea* Linn from Kashmir Himalaya. J King Saud Univ Sci. (2024) 36:103048. doi: 10.1016/j.jksus.2023.103048, PMID: 39811820

[39] Edo GI Jikah AN Onoharigho FO Akpoghelie PO Agbo JJ Ekokotu HA. The ameliorative effects of Vernonia amygdalina extract on superoxide dismutase and glutathione s-transferase on alloxan induced diabetes on male Wistar rats. Food Chem Adv. (2024) 4:100620. doi: 10.1016/j.focha.2024.100620

[40] Gadewar MM Prashanth GK Mishra PC Ashraf GM Almashjary MN Harakeh S. Evaluation of antidiabetic, antioxidant and anti-hyperlipidemic effects of Solanum indicum fruit extract in streptozotocin-induced diabetic rats. Curr Issues Mol Biol. (2023) 45:903–17. doi: 10.3390/cimb45020058, PMID: 36826003 PMC9954837

[41] Luo J Su L He X Du Y Xu N Wu R. Blood selenium and serum glutathione peroxidase levels were associated with serum β-amyloid in older adults. Biol Trace Elem Res. (2023) 201:3679–87. doi: 10.1007/s12011-022-03480-4, PMID: 36370334 PMC12001324

[42] Edo GI Ugbune U Onoharigho FO Ezekiel GO Agbo JJ. Antioxidant activities of reissantia indica willd. (mopane paddle-pod) and nephroprotective effect on paracetamol-induced nephrotoxicity in male Wistar rats. Forum Nutr. (2023) 48:26. doi: 10.1186/s41110-023-00214-x

[43] Snedecor GW Cochran WG. Statistical methods. 8th ed. Ames, IA: IOWA State University Press (1994).

[44] Chung IM Park I Seung-Hyun K Thiruvengadam M Rajakumar G. Plant-mediated synthesis of silver nanoparticles: their characteristic properties and therapeutic applications. Nanoscale Res Lett. (2016) 11:40–14. doi: 10.1186/s11671-016-1257-4, PMID: 26821160 PMC4731379

[45] Datta A Patra C Bharadwaj H Kaur S Dimri N Khajuria R. Green synthesis of zinc oxide nanoparticles using *parthenium hysterophorus* leaf extract and evaluation of their antibacterial properties. J Biotechnol Biomater. (2017) 7:271–6. doi: 10.4172/2155-952X.1000271, PMID: 37784109

[46] Rai M Ingle AP Birla S Yadav A Santos CAD. Strategic role of selected noble metal nanoparticles in medicine. Crit Rev Microbiol. (2016) 42:1–24. doi: 10.3109/1040841X.2015.1018131 26089024

[47] Venkatachalam P Kayalvizhi T Udayabanu J Benelli G Geetha N. Enhanced antibacterial and cytotoxic activity of phytochemical loaded-silver nanoparticles using *Curculigo orchioides* leaf extracts with different extraction techniques. J Clust Sci. (2017) 28:607–19. doi: 10.1007/s10876-016-1141-5

[48] Prasannaraj G Sahi SV Benelli G Venkatachalam P. Coating with active phytomolecules enhances anticancer activity of bio-engineered ag nanocomplex. J Clust Sci. (2017) 28:2349–67. doi: 10.1007/s10876-017-1227-8

[49] Gowsalya V Santhiya E Chandramohan K. Synthesis, characterization of ZnO nanoparticles from *Thespesia populnea*. Indian J Appl Res. (2017) 7:542–3.

[50] Selim YA Azb MA Ragab I Abd El-Azim HM. Green synthesis of zinc oxide nanoparticles using aqueous extract of *Deverra tortuosa* and their cytotoxic activities. Sci Rep. (2020) 10:1–9. doi: 10.1038/s41598-020-60541-1, PMID: 32103090 PMC7044426

[51] Bhuyar P Rahim MHA Sundararaju S Ramaraj R Maniam GP Govindan N. Synthesis of silver nanoparticles using marine macroalgae Padina sp. and its antibacterial activity towards pathogenic bacteria. Beni-Suef Univ J Basic Appl Sci. (2020) 9:1–15. doi: 10.1186/s43088-019-0031-y, PMID: 39806698

[52] Widatalla HA Yassin LF Alrasheid AA Ahmed SAR Widdatallah MO Eltilib SH. Green synthesis of silver nanoparticles using green tea leaf extract, characterization and evaluation of antimicrobial activity. Nanoscale Adv. (2022) 4:911–5. doi: 10.1039/D1NA00509J, PMID: 36131825 PMC9419201

[53] Yedurkar S Maurya C Mahanwar P. Biosynthesis of zinc oxide nanoparticles using *ixora coccinea* leaf extract—a green approach. Open J Synth Theory Appl. (2016) 5:1–14. doi: 10.4236/ojsta.2016.51001

[54] Muhammad W Ullah N Haroon M Abbasi BH. Optical, morphological and biological analysis of zinc oxide nanoparticles (ZnO NPs) using *Papaver somniferum* L. RSC Adv. (2019) 9:29541–8. doi: 10.1039/C9RA04424H, PMID: 35531532 PMC9071912

[55] Alsareii SA Manaa Alamri A AlAsmari MY Bawahab MA Mahnashi MH Shaikh IA. Synthesis and characterization of silver nanoparticles from Rhizophora apiculata and studies on their wound healing, antioxidant, anti-inflammatory, and cytotoxic activity. Molecules. (2022) 27:1–14. doi: 10.3390/molecules27196306, PMID: 36234841 PMC9571849

[56] Sujitha V Murugan K Paulpandi M Panneerselvam C Suresh U Roni M. Green-synthesized silver nanoparticles as a novel control tool against dengue virus (DEN-2) and its primary vector *Aedes aegypti*. Parasitol Res. (2015) 114:3315–25. doi: 10.1007/s00436-015-4556-2, PMID: 26063530

[57] Sundrarajan M Ambika S Bharathi K. Plant-extract mediated synthesis of ZnO nanoparticles using *Pongamia pinnata* and their activity against pathogenic bacteria. Adv Powder Technol. (2015) 26:1294–9. doi: 10.1016/j.apt.2015.07.001

[58] Shukla G Gaurav SS Singh A. Synthesis of mycogenic zinc oxide nanoparticles and preliminary determination of its efficacy as a larvicide against white grubs (*Holotrichia* sp.). Int Nano Lett. (2020) 10:131–9. doi: 10.1007/s40089-020-00302-0

[59] Liu Z Ren G Zhang T Yang Z. The inhibitory effects of nano-ag on voltage-gated potassium currents of hippocampal CA1 neurons. Environ Toxicol. (2011) 26:552–8. doi: 10.1002/tox.20586, PMID: 20549616

[60] Das D Mandal P. Use of biogenic silver nanoparticles in enhancing shelf life of *Morus alba* L. at post harvest stage. Sci Rep. (2020) 10:8923. doi: 10.1038/s41598-020-65953-7, PMID: 32488102 PMC7265373

[61] Ibrahim HM El-Seedy YY Gomaa NA. Cytokine response and oxidative stress status in dairy cows with acute clinical mastitis. J Dairy Vet Anim Res. (2016) 3:1–6. doi: 10.15406/jdvar.2016.03.00064

[62] Bernoud-Hubac N Lo Van A Lazar AN Lagarde M. Ischemic brain injury: involvement of lipids in the pathophysiology of stroke and therapeutic strategies. Antioxidants. (2024) 13:634. doi: 10.3390/antiox13060634, PMID: 38929073 PMC11200865

[63] Siddique NA Al-Samman AMMA. Silver nanoparticles synthesized via green chemistry with the aid of Delphinium denudatum wall. Root extract modulated gentamicin nephrotoxicity activity with respect to oxidative potential. Adv Nat Sci Nanosci Nanotechnol. (2022) 13:015003. doi: 10.1088/2043-6262/ac5497

[64] Chaitanya Kumar TV Muralidhar Y Prasad PE Prasad TNVKV Alpha RM. Evaluation of therapeutic potential of nanosilver particles synthesised using aloin in experimental murine mastitis model. IET Nanobiotechnol. (2013) 7:78–82. doi: 10.1049/iet-nbt.2012.0045, PMID: 24028805

[65] Muralidhar Y Alpha Raj M Prasad TNK Chaitanya Kumar TV Adilaxmamma K Srilatha C. Antibacterial, anti-inflammatory and antioxidant effects of acetyl-11-α-keto-β-boswellic acid mediated silver nanoparticles in experimental murine mastitis. IET Nanobiotechnol. (2017) 11:682–9. doi: 10.1049/iet-nbt.2016.0204

[66] Kiyani MM Butt MA Rehman H Ali H Hussain SA Obaid S. Antioxidant and anti-gout effects of orally administered zinc oxide nanoparticles in gouty mice. J Trace Elem Med Biol. (2019) 56:169–77. doi: 10.1016/j.jtemb.2019.08.012, PMID: 31479800

[67] Saleem A Saleem Bhat S Omonijo FA Ganai N Ibeagha-Awemu E Mudasir Ahmad S. Immunotherapy in mastitis: state of knowledge, research gaps and way forward. Vet Q. (2024) 44:1–23. doi: 10.1080/01652176.2024.2363626, PMID: 38973225 PMC11232650

[68] Chinchali JF Kaliwal BB. Effect of mastitis on mammary gland biochemical and oxidative stress parameters in experimentally induced lactating mice. Education. (2014) 4:1607–14.

[69] Zhang H Jacob JA Jiang Z Xu S Sun K Zhong Z. Hepatoprotective effect of silver nanoparticles synthesized using aqueous leaf extract of *Rhizophora apiculata*. Int J Nanomedicine. (2019) 14:3517–24. doi: 10.2147/ijn.s198895, PMID: 31190808 PMC6535432

[70] Yadav E Singh D Yadav P Verma A. Ameliorative effect of biofabricated ZnO nanoparticles of *Trianthema portulacastrum* Linn. On dermal wounds via removal of oxidative stress and inflammation. RSC Adv. (2018) 8:21621–35. doi: 10.1039/C8RA03500H, PMID: 35539937 PMC9080927

[71] Suresh D Nethravathi PC Rajanaika H Nagabhushana H Sharma SC. Green synthesis of multifunctional zinc oxide (ZnO) nanoparticles using *Cassia fistula* plant extract and their photodegradative, antioxidant and antibacterial activities. Mat Sci Semicon Proc. (2015) 31:446–54. doi: 10.1016/j.mssp.2014.12.023

[72] Ilavarasan R Vasudevan M Anbazhagan S Venkataraman S. Antioxidant activity of *Thespesia populnea* bark extracts against carbon tetrachloride-induced liver injury in rats. J Ethnopharmacol. (2003) 87:227–30. doi: 10.1016/S0378-8741(03)00147-8, PMID: 12860313

[73] Pandanaboina SC Kondeti SR Rajbanshi SL Kunala PN Pandanaboina S Pandanaboina MM. Alterations in antioxidant enzyme activities and oxidative damage in alcoholic rat tissues: protective role of *Thespesia populnea*. Food Chem. (2012) 132:150–9. doi: 10.1016/j.foodchem.2011.10.046, PMID: 26434274

[74] Jacob V Rajiv P. *In vitro* analysis: the antimicrobial and antioxidant activity of zinc oxide nanoparticles from *Curcuma longa*. Asian J Pharm Clin Res. (2019) 12:200–4. doi: 10.22159/ajpcr.2019.v12i1.28808

[75] Lakshminarayanan R Ye E Young DJ Li Z Loh XJ. Recent advances in the development of antimicrobial nanoparticles for combating resistant pathogens. Adv Healthc Mater. (2018) 7:e1701400–13. doi: 10.1002/adhm.201701400, PMID: 29717819 PMC7161883

[76] Jalilian F Chahardoli A Sadrjavadi K Fattahi A Shokoohinia Y. Green synthesized silver nanoparticle from *Allium ampeloprasum* aqueous extract: characterization, antioxidant activities, antibacterial and cytotoxicity effects. Adv Powder Technol. (2020) 31:1323–32. doi: 10.1016/j.apt.2020.01.011

[77] Min H Arturo AK Xiaomi W Liyan T Bing W Rong J. Concentrations of silver nanoparticles and silver ions perturb the antioxidant defense system and nitrogen metabolism in N_2_-fixing Cyanobacteria. Environ Sci Technol. (2020) 54:15996–6005. doi: 10.1021/acs.est.0c05300, PMID: 33232140

